# Potential Biases in Estimating Absolute and Relative Case-Fatality Risks during Outbreaks

**DOI:** 10.1371/journal.pntd.0003846

**Published:** 2015-07-16

**Authors:** Marc Lipsitch, Christl A. Donnelly, Christophe Fraser, Isobel M. Blake, Anne Cori, Ilaria Dorigatti, Neil M. Ferguson, Tini Garske, Harriet L. Mills, Steven Riley, Maria D. Van Kerkhove, Miguel A. Hernán

**Affiliations:** 1 Center for Communicable Disease Dynamics, Department of Epidemiology, Harvard T.H. Chan School of Public Health, Boston, Massachusetts, United States of America; 2 Department of Immunology and Infectious Diseases, Harvard T.H. Chan School of Public Health, Boston, Massachusetts, United States of America; 3 MRC Centre for Outbreak Analysis and Modelling, Department of Infectious Disease Epidemiology, Imperial College London, London, United Kingdom; 4 Centre for Global Health, Institut Pasteur, Paris, France; 5 Department of Biostatistics, Harvard T.H. Chan School of Public Health, Boston, Massachusetts, United States of America; Yale University, UNITED STATES

## Abstract

Estimating the case-fatality risk (CFR)—the probability that a person dies from an infection given that they are a case—is a high priority in epidemiologic investigation of newly emerging infectious diseases and sometimes in new outbreaks of known infectious diseases. The data available to estimate the overall CFR are often gathered for other purposes (e.g., surveillance) in challenging circumstances. We describe two forms of bias that may affect the estimation of the overall CFR—preferential ascertainment of severe cases and bias from reporting delays—and review solutions that have been proposed and implemented in past epidemics. Also of interest is the estimation of the causal impact of specific interventions (e.g., hospitalization, or hospitalization at a particular hospital) on survival, which can be estimated as a relative CFR for two or more groups. When observational data are used for this purpose, three more sources of bias may arise: confounding, survivorship bias, and selection due to preferential inclusion in surveillance datasets of those who are hospitalized and/or die. We illustrate these biases and caution against causal interpretation of differential CFR among those receiving different interventions in observational datasets. Again, we discuss ways to reduce these biases, particularly by estimating outcomes in smaller but more systematically defined cohorts ascertained before the onset of symptoms, such as those identified by forward contact tracing. Finally, we discuss the circumstances in which these biases may affect non-causal interpretation of risk factors for death among cases.

The case-fatality risk (CFR) is a key quantity in characterizing new infectious agents and new outbreaks of known agents. The CFR can be defined as the probability that a case dies from the infection. Several variations of the definition of “case” are used for different infections, as discussed in Box 1. Under all these definitions, the CFR characterizes the severity of an infection and is useful for planning and determining the intensity of a response to an outbreak [1,2]. Moreover, the CFR may be compared between cases who do and do not receive particular treatments as a way of trying to estimate the causal impact of these treatments on survival. Such causal inference might ideally be done in a randomized trial in which individuals are randomly assigned to treatments, but this is often not possible during an outbreak for logistical, ethical, and other reasons [3]. Therefore, observational estimates of CFR under different treatment conditions may be the only available means to assess the impact of various treatments.

**Box 1. Definition of the CFR.**

The CFR itself is an ambiguous term, as its definition and value depend on what qualifies an individual to be a “case.” Several different precise definitions of CFR have been used in practice, as have several imprecise ones. The infection-fatality risk (sometimes written IFR) defines a case as a person who has shown evidence of infection, either by clinical detection of the pathogen or by seroconversion or other immune response. Such individuals may or may not be symptomatic, though asymptomatic ones may go undetected. The symptomatic case-fatality risk (sCFR) defines a case as someone who is infected and shows certain symptoms. Infection in many outbreaks is given several gradations, including confirmed (definitive laboratory confirmation), probable (high degree of suspicion, by various clinical and epidemiologic criteria, without laboratory confirmation), and possible or suspected (lower degree of suspicion). This paper describes issues in estimating any of these risks or comparing them across groups, but does not go into the details of each possible definition.

Furthermore, unlike risks commonly used in epidemiologic research (e.g., the 5-year mortality risk), the length of the period during which deaths are counted for the CFR is rarely explicit, probably because it is considered to be short enough to avoid ambiguity in the definition of CFR. However, a precise definition of the CFR would need to include the risk period, e.g., the 1-month CFR of Ebola. Clearly, the definition of CFR for a particular investigation should be specified as precisely as possible.

However, observational studies conducted in the early phases of an outbreak, when public health authorities are appropriately concentrating on crisis response and not on rigorous study design, are challenging. A common problem is that disease severity of the cases recorded in a surveillance database will differ, perhaps substantially, from that of all cases in the population. This issue has arisen in the present epidemic of Ebola virus disease in West Africa and in many previous outbreaks and epidemics [4–9] and will continue to arise in future ones.

Here we outline two biases that may occur when estimating the CFR in a population from a surveillance database, and three more biases that may occur when comparing the CFR between subgroups to estimate the causal effect of medical interventions. We also briefly consider the applicability of these biases to a different application: comparing the CFR across different groups of people, for example, by geography, sex, age, comorbidities, and other “unchangeable” risk factors. Such factors are “unchangeable” in the sense that they are not candidates for intervention in the setting of the outbreak, though some could, of course, change over longer timescales. The goal of estimating the CFR in groups defined by such unchangeable factors is not to understand the causal role of these factors in mortality, but to develop a predictive model for mortality that might be used to improve prognostic accuracy or identify disparities. Such predictions may be affected by survivorship bias and selection bias, but not by confounding, as we discuss.

## Biases Affecting the Estimation of the Overall CFR

Two biases that may affect the estimation of an overall CFR are presented in Table 1:

**Table 1:** Potential biases that can affect the estimation of CFR (and thereby also the comparison of CFR across groups).

Bias	Direction	Outbreaks in which analysts have noted this bias may be operating	Possible solutions
**Preferential ascertainment of severe cases:** In an infection with a range of manifestations from relatively mild to highly severe, milder cases are less likely to appear in surveillance databases than more severe ones; therefore, the CFR among ascertained cases will be higher than that among all cases.	Spuriously increases estimate of CFR	Influenza H1N1pdm [10–12], Influenza H7N9 [6], Influenza H5N1 [7] (though this hypothesis has been refuted [8]), Middle East Respiratory Syndrome [4], Ebola (this article)[13]	Note: these solutions are listed in approximately the temporal order in which they may be practical, from early in the outbreak to later on; details will depend on the epidemiology of the outbreak.
			Use sentinel surveillance sites to estimate multipliers between various levels of severity and extrapolate to a larger population [6].
			Survey- or health-facility–based surveillance for symptomatic infection [14] in a defined population, combined with enhanced surveillance for severe outcomes (particularly death) in the same population.
			Use travelers from high-burden areas with low ascertainment to low-burden areas with higher ascertainment to estimate incidence of infection in source population [15,16], thereby providing a more accurate denominator for comparison to deaths in source population.
			Surveillance pyramid approaches: reconstruct conditional probabilities of appearing at one severity level conditional on reaching a lower severity level; combine data sources that have relatively complete ascertainment of higher severity levels (e.g., hospitalization, ICU, death) with those having relatively complete ascertainment of lower levels (e.g., seeking medical attention, hospitalization) [10,11]. CFR can then be estimated as a product of conditional probabilities with associated uncertainties [17].
			Serologic ascertainment of infection [18,19] to provide a population denominator for infections regardless of symptoms, combined with active surveillance for more severe outcomes.
			Individuals ascertained by a different mechanism, e.g., named healthy contacts of cases who subsequently test positive, could be a more representative group in whom to assess severity [20].
**Bias due to delayed reporting.** During an ongoing epidemic, at any week *w* the persons who have died up to time *w* will not be the only ones to die of the infection among those who became cases by *w*. The denominator of the CFR (cases) includes persons who have not yet died of the infection, but will do so in the future. Thus the CFR by *w* will be less than the true CFR. This bias will be particularly severe for infections that are increasing rapidly in incidence and for which the infection–death time interval is long.	Spuriously decreases estimate of CFR	SARS [9], Influenza H1N1pdm [21], Ebola [22,23]	Limit analysis to those cases with sufficiently long follow-up for a death to have been recorded had a death occurred. While this may lead to extremely small sample sizes near the beginning of an epidemic, this strategy is more feasible after a local epidemic wave, including reporting delays, has passed or nearly passed [10,11].
			Limit analysis to those cases known either to have died or recovered, but exclude those with unknown outcome (biased if severity affects outcome ascertainment)[22–24].
			Apply a competing-risk Kaplan-Meier–like method or a parametric mixture model to the full dataset (biased if the times to death and time to recovery have different distributions) [24,25].
			Fit the distribution of times to death and to recovery to estimate the true CFR [10,11], or inverse-probability weight deaths using the conditional probability of having survived by w, given that one dies [21] (biased if the probability distribution is incorrect).

### Preferential ascertainment of severe cases

For diseases that have a spectrum of clinical presentation, those cases that come to the attention of public health authorities and are entered into surveillance databases will typically be people with the most severe symptoms, who seek medical care, are admitted to hospital, or die. Therefore, the CFR will typically be higher among detected cases than among the entire population of cases, given that the latter may include individuals with mild, subclinical, and (under some definitions of “case”) asymptomatic presentations. Laboratory confirmation as an inclusion criterion may reduce this bias if it is able to detect a wider spectrum of presentations, or may exacerbate it if the probability of receiving a laboratory test is higher for more severe cases and/or if test sensitivity is higher for more severe cases. The magnitude of this bias may be uncertain for a long period because the spectrum of clinical presentations is itself uncertain at the start of an outbreak of a new disease [12,26]. All proposed approaches to estimate and correct for this bias (Table 1) require auxiliary data sources to estimate how the reported subset of cases compares with the overall population of cases. The availability of such auxiliary data sources will depend on the context of the outbreak.

### Bias due to delayed reporting of death

**Fig 1:**
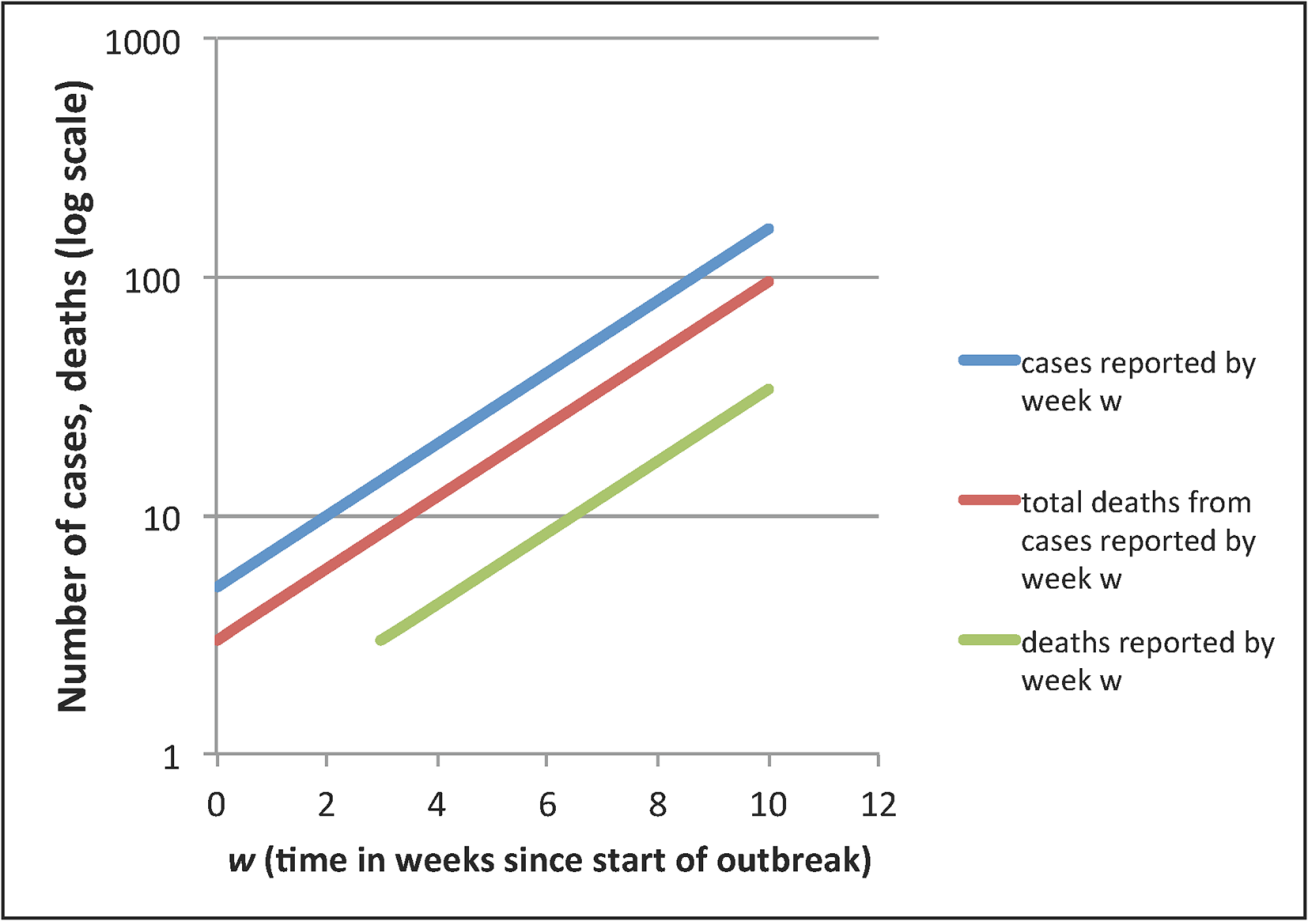
Illustration of delayed reporting bias in an exponentially growing epidemic. In an ongoing epidemic, there will typically be a delay between the reporting of a case and the reporting of the death of that case, if the infected person dies. Thus, at any moment, there will be some cases reported who will die of the infection but who have not yet died, or whose deaths have not yet been reported. Simple division of the number of deaths reported by week *w* (green), by the number of cases reported by week *w* (blue) will underestimate the CFR because the numerator does not include all those cases in the denominator who will eventually die. With a reporting delay of 3 weeks for deaths compared to cases, the reported deaths curve will be shifted 3 weeks to the right, relative to the curve of the total number of cases reported by week *w* who will die (red). If the epidemic doubling time is 2 weeks, as shown here, the underestimate of CFR will be by a factor of about 2^3/2^ ≈ 2.8, with the exponent being the number of epidemic doubling times that pass between case reporting and death reporting. In reality, there will be a distribution of reporting delays rather than a fixed delay, making this a heuristic rather than exact approach. The problem is ameliorated in an epidemic that grows more slowly or less than exponentially. For more details, see references in Table 1.

During an ongoing epidemic, there is a delay between the time someone dies and the time their death is reported. Therefore, at any moment in time, the list of cases includes people who will die and whose death has not yet occurred, or has occurred but not yet been reported. Thus dividing the cumulative number of reported deaths by the cumulative number of reported cases at any moment will underestimate the true CFR. The key determinants of the magnitude of the bias are the epidemic growth rate and the distribution of delays from case-reporting to death-reporting; the longer the delays and the faster the growth rate, the greater the bias. Heuristically, the underestimate will be proportionate to the expansion of the epidemic during the delay between the time a case enters the database to the time the death of that case enters the database (if it occurs). Fig 1 illustrates an example where the delay is 3 weeks, the epidemic doubling time is 2 weeks, and the underestimate is by a factor of 2^3/2^ ≈ 2.8.

This bias may be corrected for in various ways, and to varying degrees, using information on the growth rate of the epidemic, the distribution of times from case-report to death-report, and the distribution of times from case-report to recovery-report (i.e., report that the case is no longer at risk of dying of the infection). A simple approach is to limit analysis to those cases with sufficiently long follow-up for a death to have been recorded had a death occurred, but this approach may result in an exceedingly small sample size if applied early in the epidemic. Several such strategies are described in Table 1.

## Biases Affecting the Causal Interpretation of Relative CFR

Here, and in Table 2, we discuss the sources of three biases that threaten the validity of a causal interpretation of a difference in CFR between groups who have received different interventions. Such a difference might be measured as a risk ratio (RR), the ratio of CFR in group A to that in group B, or as an odds ratio (OR), the ratio of the odds of dying in group A and group B, or as a risk difference (RD), the difference between the CFR in group A and group B. We use the term relative CFR to refer to any of these measures, and call a relative CFR non-null when it differs from 1 (ratio) or 0 (difference).

**Table 2:** Potential biases that can affect the comparison of CFR across groups (relative CFR), using the example of comparing the CFR among hospitalized and non-hospitalized persons to assess the relative CFR for hospitalization.

Bias	Direction	Outbreaks in which analysts have noted this bias may be operating	Possible solutions/means of detecting the bias
**Survivorship bias:** Those who die before being hospitalized cannot, by definition, be hospitalized; a crude comparison of deaths among hospitalized and non-hospitalized cases will therefore reflect the “protective” effect of death against hospitalization. This is an example of reverse causality because for these individuals, death prevented hospitalization, rather than hospitalization preventing death.	Spurious protective effect of hospitalization on risk of death	Ebola (this article)	Conditioning analysis on survival up to day *d* of symptoms, and analyzing hospitalization on day *d* as the intervention, will avoid this bias, as individuals who die before hospitalization will not be included in the analysis. This analysis can be repeated for different values of *d* and potentially combined in a parametric model.
			Individuals identified before becoming cases (e.g., as healthy contacts of infected persons) and actively followed regardless of clinical severity could be analyzed separately as a prospective cohort for whom the course of disease could be observed and this restriction readily made.
**Confounding:** if individuals are hospitalized in response to predictors of poor prognosis, hospitalization will be noncausally associated with poor outcome. This problem is common in the pharmacoepidemiology literature [27]. Alternatively, in situations of triage, when beds or other resources are limited, individuals with better prognosis may receive hospitalization (or another intervention), creating a spurious beneficial effect of hospitalization.	May be in either direction, depending on whether those receiving the intervention have better or worse prognosis.	Ebola (this article), H1N1pdm (effect of antiviral treatment on death) [28], Influenza H5N1 (effect of antiviral treatment on death) [29,30,31]	In principle, analysis can adjust for prognostic factors that also predict hospitalization via matching, stratification, or multivariable analysis. In practice, such information may be unavailable [27].
			Such adjustments will be more readily made if data are obtained prospectively from a cohort of cases identified before becoming cases.
**Selection bias occurring because mortality and hospitalization both affect the probability a case will appear in the database [32]:** When inclusion in a database can occur as a result of either of two (or more) factors, the association between these two factors within the database will be biased relative to that in the source population. For example, if death and hospital admission are both means by which cases are ascertained and enter a database, as may be the case for Ebola datasets, hospitalization will be spuriously associated with death in the dataset even if hospitalization has no causal effect in preventing death.	Direction of bias depends on the probabilities of inclusion in the dataset depending on exposure and outcome.	Ebola (this article)	Without knowledge of how cases came to enter a dataset, the magnitude of this bias cannot be evaluated. Under assumptions about the proportion of cases entering the dataset for various reasons, a sensitivity analysis could be performed to assess the plausibility of assigning any observed protective effect to this bias [33].
			This bias too may be avoided by prospectively following a cohort of individuals who are identified before becoming cases.

When these biases are present, a relative CFR, different from the null value in group B compared with A does not imply a causal effect of group. For example, if group A is non-hospitalized patients and group B is hospitalized patients, an odds ratio of death less than 1 may not imply a beneficial effect of hospitalization on the odds of death. Similarly, a relative CFR greater than 1 may not imply that hospitalization is harmful. We use the estimation of the causal impact of hospitalization on mortality as our example throughout this section. Note that exactly the same reasoning applies to assessment of another intervention or to a comparison of two interventions, for example, a comparison of treatment at Center A versus treatment at Center B.

The first bias arises in a naïve comparison of mortality between those who have and those who have not been hospitalized. If some individuals die before they can be admitted to a hospital, they will by definition not become hospitalized. Therefore, even in the absence of any effect of hospitalization on the risk of death, there will be fewer deaths among those hospitalized than among those not hospitalized. We will refer to this bias as “survivorship bias.”

This bias can be eliminated using data on the time *d* since the person became a case. The analysis would then compare the risk of death between those individuals who became hospitalized on day *d* against those who did not, limiting analysis to those who were still alive at day *d*. This estimate might be expected to differ for different values of *d* if, for example, early hospitalization was more beneficial than late hospitalization. If not different, the estimates of the causal effect of hospitalization for several values of *d* could be combined. Restriction to those alive at the time from which we want to estimate the effect of hospitalization would eliminate survivorship bias. However, two other biases, described below, could still affect the inference.

The second source of bias is confounding. Severity of disease will likely affect the probability of hospitalization and the probability of death. As a common cause of the exposure of interest (hospitalization) and the outcome (death), disease severity is a confounder of the causal effect of hospitalization on death. If hospitalization is offered to especially severe cases or—in the setting of extreme triage—to especially mild cases, then hospitalization would spuriously appear harmful (if hospitalization went to especially severe cases) or beneficial (if it went to especially mild cases). There may be other confounders of this effect besides disease severity. Individuals living in rural areas may be at greater risk of mortality (e.g., due to malnutrition) and also less likely to be hospitalized (due to longer travel time to hospital). Place of residence (or travel time to hospital) in this setting would be a confounder of the effect of hospitalization on death. The standard approach to reducing confounding is to stratify, restrict, or adjust for prognostic factors that affect the propensity to receive the treatment (in this case to be hospitalized) [27]. However, such information may frequently be limited or unavailable in databases compiled during outbreaks, especially in resource-limited settings.

The third source of bias is selection occurring because mortality and hospitalization both affect the probability a case will appear in the database. During an outbreak, many cases may not appear in the database because they are not ascertained or because information about them is not obtainable. In particular, cases who are not hospitalized, and cases who do not die, may be less likely than other cases to appear in the database because they are less likely to come to medical or public health attention.

If appearance in a database is the common effect of hospitalization and death, then the association between hospitalization and death among cases in the database may be non-null even if hospitalization and death were independent in the population of all cases. The direction and magnitude of the association between hospitalization and death among cases in the database will then be the result of combining the association due to this selection bias, the association due to a potential effect of hospitalization on mortality, and the association due to confounding.

Hypothetical examples are shown in Tables 3–5. In these tables, the association in the population between hospitalization on day 8 (an arbitrarily chosen day) and death is negative; individuals hospitalized on day 8 (an arbitrarily chosen day) of symptoms have a lower probability of death than those who are not hospitalized on day 8 of symptoms. If we assume that this analysis has avoided survivorship bias by limiting analysis to cases still alive on day 8, then the population-level association would reflect a combination of the causal effect of hospitalization on day 8 on risk of death, and confounding by severity or other factors. This population-level association is the same in Tables 3, 4, and 5, but different probabilities are assumed for inclusion in the database, depending on whether an individual is hospitalized on day 8, dies, or both. Relative CFRs on the RR, OR, and RD scales for hospitalization on day 8 are calculated for each hypothetical example.

**Table 3:** Effect of selection bias on estimates of relative CFR on the risk ratio (RR) and odds ratio (OR) scale.

Joint frequencies of hospitalization and death in the whole population among those alive at day 8 of symptoms					
	Not hospitalized on day 8 of symptoms	Hospitalized on day 8 of symptoms	Total		
Survive	200	400	600	*RR*_*P*_ =	0.75
Die	800	600	1,400	*OR*_*P*_ =	0.375
Total	1,000	1,000	2,000	*RD*_*P*_ =	-0.20
Assumed probability of being in the database sample given hospitalization and death					
	Not hospitalized on day 8 of symptoms	Hospitalized on day 8 of symptoms	Average		
Survive	5%	40%	28%	*OR*_*S*_ =	0.16
Die	40%	50%	44%		
Average	33%	46%	40%		
Frequencies of persons in the database					
	Not hospitalized on day 8 of symptoms	Hospitalized on day 8 of symptoms	Total		
Survive	10	160	170	*RR*_*D*_ =	0.67
Die	320	300	620	*OR*_*D*_ =	0.06
Total	330	460	790	*RD*_*D*_ =	-0.32

Subscript P represents the population values, while subscript D represents the values measured for those cases included in the data base; selection bias produces the discrepancy. The extent of selection bias may be measured as $OR_{s}=\frac{S_{00}S_{11}}{S_{01}S_{10}}$, where *S*_*ij*_ is the probability a case with exposure (hospitalization at day 8) i and outcome (mortality) j appears in the database. In this example, selection bias spuriously enhances the negative association between hospitalization on day 8 and death, on all scales: RR, OR, and RD.

**Table 4:** Effect of selection bias on estimates of relative CFR on the risk ratio (RR) and odds ratio (OR) scale.

Joint frequencies of hospitalization and death in the whole population among those alive at day 8 of symptoms					
	Not hospitalized on day 8 of symptoms	Hospitalized on day 8 of symptoms	Total		
Survive	200	400	600	*RR*_*P*_ =	0.75
Die	800	600	1,400	*OR*_*P*_ =	0.375
Total	1,000	1,000	2,000	*RD*_*P*_ =	-0.20
Assumed probability of being in the database sample given hospitalization and death					
	Not hospitalized on day 8 of symptoms	Hospitalized on day 8 of symptoms	Average		
Survive	20%	40%	33%	*OR*_*S*_ =	0.88
Die	40%	70%	53%		
Average	36%	58%	47%		
Frequencies of persons in the database					
	Not hospitalized on day 8 of symptoms	Hospitalized on day 8 of symptoms	Total		
Survive	40	160	200	*RR*_*D*_ *=*	0.81
Die	320	420	740	*OR*_*D*_ =	0.33
Total	360	580	940	*RD*_*D*_ =	-0.16

In this example, selection bias spuriously enhances the negative association between hospitalization on day 8 and death on the RR and RD scales and reduces it (biases toward a null association) on the OR scale.

**Table 5:** Effect of selection bias on estimates of relative CFR on the risk ratio (RR) odds ratio (OR) and risk difference (RD) scales.

Joint frequencies of hospitalization and death in the whole population among those alive at day 8 of symptoms					
	Not hospitalized on day 8 of symptoms	Hospitalized on day 8 of symptoms	Total		
Survive	200	400	600	*RR*_*P*_ =	0.75
Die	800	600	1,400	*OR*_*P*_ =	0.375
Total	1,000	1,000	2,000	*RD*_*P*_ =	-0.20
Assumed probability of being in the database sample given hospitalization and death					
	Not hospitalized on day 8 of symptoms	Hospitalized on day 8 of symptoms	Average		
Survive	20%	40%	33%	*OR*_*S*_ =	1.13
Die	40%	90%	61%		
Average	36%	70%	53%		
Frequencies of persons in the database					
	Not hospitalized on day 8 of symptoms	Hospitalized on day 8 of symptoms	Total		
Survive	40	160	200	*RR*_*D*_ =	0.87
Die	320	540	860	*OR*_*D*_ =	0.42
Total	360	700	1060	*RD*_*D*_ =	-0.12

In this example, selection bias spuriously reduces the negative association between hospitalization on day 8 and death, on all three scales: RR, OR, and RD.

The hypothetical data in these tables show that selection bias in such a circumstance may be either positive or negative on each of the three scales, depending on the specific probabilities of selection in each of the four states. Table 3 shows an example of negative bias on the RR, OR, and RD scales (overestimating the protective effect of hospitalization on day 8 expressed as a lower value of each relative risk measure). Table 4 shows an example of a positive bias on the RR and RD scales and a negative bias on the OR scale. Table 5 shows an example of positive bias (underestimating the protective effect of hospitalization on day 8 expressed as a higher value of each measure) on all three scales.

From experience, it seems that when databases are assembled in this way, it is rarely possible to tell why an individual case has come into the database. In the absence of such information, it is difficult to imagine how adjustments could be performed. However, sensitivity analyses could be performed to assess how strong such biases are likely to be [33].

## Addressing the Biases in Causal Interpretation of Relative CFR

We have stated already that survivorship bias can be avoided by limiting analyses of the intervention to those who remain alive on a certain day after becoming a case. One strategy that would help to resolve the other two sources of bias is to limit analysis to a cohort of cases who were identified before they became cases; for example those who were identified as healthy contacts of known cases, and were followed prospectively. Confounding occurs because individual factors like severity of infection or place of residence (which could affect both the probability of exposure—receiving the intervention—and the probability of the outcome—mortality) are not accounted for in the analysis through stratification, restriction, or adjustment. Selection bias in this setting occurs because the exposure and the outcome both affect the probability of inclusion in the database. Follow-up of a cohort of contacts ascertained before becoming cases could eliminate hospitalization and mortality as predictors of inclusion in the database, thus eliminating the form of selection bias we have discussed. It would provide an opportunity for gathering data on severity and other predictors of exposure and outcome, which would facilitate control of confounding, though not guarantee to eliminate it. Such a cohort would also provide a natural setting for analyses that avoid survivorship bias. The cost of such improvements in inference would be the need to ascertain such contacts and maintain surveillance of those individuals, following them to obtain data on relevant covariates. Such a strategy–which has been followed in cases of exposed health-care workers in settings with high resources and few cases—would likely have benefits for the individuals followed (e.g., increasing the probability they receive care if infected) and for reducing transmission (if such individuals were promptly isolated upon evidence of infection). However, it has not been possible so far in the large Ebola outbreaks in West Africa to do this routinely.

## Biases in Predicting Outcomes without Causal Interpretation

It is often of interest to predict the probability of mortality for an individual case of an infectious disease based on that individual’s demographic and clinical data, without placing any causal interpretation on the factors used to predict outcome. For example, in 2009, there was much interest in whether morbid obesity (or obesity in general) was predictive of worse outcome in infection with the novel pandemic strain of influenza A/H1N1 [34].The primary goal was to improve estimates of clinical prognosis, although observations about prognosis could later be used to generate causal hypotheses for further testing. Similarly, observations of disparate rates of severe outcomes by geography within New York City did not initially involve causal judgments about why certain areas had worse outcomes, although they could be used to guide enhancement of services in areas with worse outcomes [35]. Even for a well-understood disease like polio, it may be necessary to identify unusual demographic patterns of mortality in order to understand and respond effectively to an outbreak [36].

Prognostic exercises such as these cannot suffer from confounding bias because no causal interpretation is attached to the conclusions. They can, however, suffer from selection bias. Returning to the Ebola context, one might wish to know whether pregnant women infected with Ebola are at greater risk of death from Ebola infection than other cases [37], for example, in order to give them greater supportive care. If the probability of entering the database depends on whether an Ebola patient is pregnant and on whether she ultimately dies of the infection, then the probability of death given pregnancy will likely differ in the database from the value in the population of direct interest for a clinical or public health decision maker. If the goal of analysis is to inform public health decision makers on the value of efforts to prevent infection in pregnant women, then the population-wide CFR among pregnant women is the value of direct interest. If, on the other hand, the goal of analysis is to inform health care providers at a treatment center to make a better clinical decision based on an accurate prognosis of the patient presenting to them, the quantity of direct interest is the probability of death among pregnant women in the population they encounter—those admitted to the treatment center. This value, again, will differ from that in the database, which may (in our running example) have been enriched for individuals entered in the database because they died of the infection. It will also differ from that in the overall population. The general point is that selection bias can be operative if the population on which analysis is performed is not a representative sample of the population for which the value of the CFR is sought, and selection bias of this form can lead to spurious conclusions in prognostic estimates as well as in causal ones. As in the case of causal inference, prognostic estimates will avoid selection bias to the extent they can be performed on a randomly chosen cohort of cases, identified via tracing of healthy contacts, for example.

## Discussion

To determine the appropriate scope and magnitude of public health response to an infectious disease outbreak, it is important to estimate the CFR and the determinants of its variation [1,5]. For example, in the 2009 influenza pandemic, early point estimates of the CFR ranged over orders of magnitude, from a value below that of seasonal influenza, which would have justified a modest response, to values around 1%, approximately half that of the 1918 pandemic, which would have indicated the need for massive interventions to protect public health [12,15–17,21]. To a large degree, this variation reflected judgments that one or the other of the biases in Table 1 was more important, judgments that were difficult to make accurately and confidently on the rapid timescale required for decision making [26].

In other situations, accurate assessment of the CFR is not as crucial for decisions about the scale of response required; for example, in the ongoing 2014 Ebola epidemic in West Africa, the uncertainty about the CFR is limited to a range between high values and very high values, and it is not clear that any greater response would be indicated by a 90% CFR than a 60% CFR [22]. Either way, a rapid and massive response is warranted.

Even when the overall CFR is not a key input to decision making, there is obvious value to inferences about which conditions lead to a lower CFR, whether these be specific treatment, particular types of supportive care, or hospitalization in general. Moreover, treatment facilities might be evaluated by the proportion of their patients who survive; here the relative CFR calculated would be for treatment in one facility versus treatment in another. There will be a temptation to conclude that treatment facilities with higher CFR are doing a worse job—that is, to apply a causal interpretation to observed differences in the CFR. Even in settings with more resources to measure covariates, methods of risk-adjustment of comparative outcomes to account for the mix of patients seen are complex and controversial [38]. In an emergency setting, with few covariates available to characterize the “case mix” of a health care provider, causal interpretation of differences in CFR would be particularly prone to error, potentially producing conclusions that mislead and thereby damage control efforts. For instance, if through confounding, larger referral treatment centers primarily receive patients who have survived infection for some time and are therefore less likely to die, independently of treatment, this may be erroneously interpreted as more effective treatment in these centers. Similarly, if certain treatment centers preferentially admit the most symptomatic patients, they may falsely appear to be less effective or even harmful to patient outcome. With at least five separate sources of bias in CFR or relative CFR estimates, and only imperfect solutions typically available for most due to lack of data, separating causal from non-causal factors in relative CFR estimation seems extremely risky. This is not to deny that data should be gathered or analyzed; on the contrary, the biases here suggest that more thorough data gathering is necessary before analyses of such quantities as relative CFR are relied upon for any decision.

There has been much debate, particularly in the area of Ebola treatments, about whether randomized studies comparing a treatment to a placebo are ethical [3,39]. Whatever one’s view on this debate, it seems likely that some observational (non-randomized) studies of the effectiveness of particular therapies, or the comparative effectiveness of two or more therapeutic approaches will occur, whether for ethical reasons, logistical reasons, or both. Such studies—in which a key endpoint will be mortality—will be vulnerable to the sorts of biases described in this article, particularly in cases in which the true effect size of the treatment is limited. The biases described here should be kept in mind when evaluating the conclusions of such studies, and wherever possible, studies should be designed to minimize them. Small studies conducted using systematic approaches to enrollment and follow-up of patients may be more precise and less biased than studies with larger sample sizes that use databases collected for other reasons. Similarly, there may be situations in which efforts are made to administer scarce therapeutic agents to those most likely to benefit from them. Such efforts rely on estimates, formal or informal, of the prognosis of patients with and without the treatment, depending on variables such as the time since they became symptomatic. These estimates, too, may be affected by the biases discussed.

In the current Ebola outbreak in West Africa, such data gathering has not routinely occurred, for a number of reasons, including lack of health system infrastructure [40] and prioritization of crisis response and other directly lifesaving activities. In future outbreaks of other diseases, as in the past with pandemic influenza, setting up systematic approaches to gather data useful for such assessments should be a priority [1,5]. Meanwhile, emphasis on recording for each patient in a database the time, place, and circumstances (e.g., hospital, clinic, funeral, contact tracing) under which the information is being gathered can substantially improve our ability to account for biases induced by a database with unplanned entry criteria.

To reduce the impact of the biases identified on causal and (where applicable) prognostic inference, it appears desirable when possible to limit analysis to a subset of cases who have been followed prospectively since they became cases. These individuals might most likely be identified by forward contact tracing, in which cases are asked to name healthy individuals with whom they have had contact, and those individuals are followed to identify further infections. It has previously been noted that cases identified by contact tracing are more representative of cases in the general infected population than those identified because of symptoms, medical need, or death [20,41]. Use of such a sample does not guarantee to eliminate biases, as there may be residual confounding not adequately controlled in the analysis or subtler forms of selection bias (e.g., differential loss to follow up within the sample) [32], but should significantly reduce them.

We have emphasized the relevance of several biases to interpretation of datasets gathered in an emergency, such as the early phases of an emerging infection. While the downward bias in estimation of the CFR due to delayed reporting of deaths is most acute in rapidly growing epidemics, the other biases described may apply regardless of the overall trajectory of an epidemic, and thus may apply to endemic diseases as well as emerging ones. Nonetheless, due to the sense of urgency to gather data and scale-up a response simultaneously, datasets assembled during infectious disease outbreak or emergency settings are especially prone to include unplanned mixes of cases who enter the dataset for various reasons. Biases of the sorts described here should be systematically considered whenever one attempts to extract causal inferences from such observational data, and alternative, more systematic data collection should be considered when possible.

**Key Learning Points**

- Datasets available at the onset of new epidemics of infectious diseases are often collected for reasons other than epidemiologic analysis of absolute and comparative case-fatality risks (CFR), and estimates of such quantities based on these data may be subject to biases, the relative magnitudes of which are difficult to ascertain and vary by situation.
- Major sources of bias affecting the estimation of absolute CFR are differences in severity between all cases and the subset of cases who enter the dataset, typically leading to inflated estimates of CFR, and more rapid reporting (less delay) in reporting cases than in reporting the deaths of those cases, typically leading to underestimates of CFR.
- Biases affecting the causal interpretation of relative CFR (causal attribution of different CFR in different groups to a particular intervention in one group, e.g., hospitalization) may arise from survivorship bias, in which individuals who survive longer may be more likely to receive the intervention; from confounding, in which a common factor (e.g., disease severity) affects the probability of both the intervention and mortality; and from selection bias, in which individuals are more or less likely to enter the dataset as a function of whether they receive the intervention and whether they have the outcome.
- These biases may be severe enough to lead to qualitatively mistaken inferences about the severity of the infection or about the impact of interventions (such as hospitalization) on mortality, and may be particularly misleading when comparing, for example, the effect of hospitalization at different centers, given that cases hospitalized at different centers may enter the dataset for different reasons.
- Methods exist to identify and reduce these biases. In particular, the use of small but carefully defined cohorts of individuals who are followed from the time of infection or symptom onset (perhaps those identified via contact tracing) may ameliorate many of these biases.

**Top Five Papers**

1. Donnelly CA, Ghani AC, Leung GM, Hedley AJ, Fraser C, Riley S, et al. Epidemiological determinants of spread of causal agent of severe acute respiratory syndrome in Hong Kong. Lancet. 2003 May 24;361(9371):1761–6.
2. Greenland S. Basic methods for sensitivity analysis of biases. Int J Epidemiol. 1996 Dec;25(6):1107–16.
3. Garske T, Legrand J, Donnelly CA, Ward H, Cauchemez S, Fraser C, et al. Assessing the severity of the novel influenza A/H1N1 pandemic. BMJ. 2009 Jul 14;339:b2840.
4. Hernan MA, Hernandez-Diaz S, Robins JM. A structural approach to selection bias. Epidemiology. 2044;15:615–25.
5. Lipsitch M, Finelli L, Heffernan RT, Leung GM, Redd SC, 2009 H1N1 Surveillance Group. Improving the evidence base for decision making during a pandemic: the example of 2009 influenza A/H1N1. Biosecur Bioterror. 2011 Jun;9(2):89–115.
